# A call for mentorship in otolaryngology^☆^

**DOI:** 10.1016/j.bjorl.2018.10.005

**Published:** 2018-11-08

**Authors:** Peter H. Hwang

**Affiliations:** Stanford University School of Medicine, Department of Otolaryngology-Head & Neck Surgery, Stanford, United States

Mentorship matters. The guidance, wisdom, and benevolence of a more experienced colleague is a precious gift that can positively influence the trajectory of one's career. It is likely that among this Journal's readership, those of us who have been in practice for at least a decade can recall a few key individuals in our life who selflessly donated their time to serve as mentors. We can probably appreciate the specific ways that our careers were influenced or transformed by the wisdom of our mentors. Yet, for all of the positive thoughts surrounding mentorship, strong mentors seem to be in perennially short supply. For those of us who have been the beneficiaries of committed mentorship, our collective memory for the importance of being mentored tends to fade when we are called on to mentor others – too many patients to care for, not enough hours in the week, and so on. And yet we also recognize that we likely would not be where we are in our careers had it not been for the generosity of others who carved out time from their busy schedules for our sake.

The importance of mentorship in academic medicine has been validated by research in the field. Having a mentor has been associated with significantly higher career satisfaction scores, greater research productivity, and shorter time to promotion.^1^, ^2^, ^3^ Research has also elucidated the essential qualities of the successful mentor: professionally, the successful mentor is collaborative, intellectual, and well connected; personally, the successful mentor is altruistic, compassionate, honest and non-judgmental.^4^, ^5^ Research has also identified the qualities of the successful mentee: committed, open to feedback, self-appraising, and actively listening.^4^

Once the successful pairing of mentor to mentee has taken place, the relationship requires mutual commitment, respect, and enthusiasm in order for it to thrive. The relationship should be mentee-focused, with the agenda driven by the mentee, who needs to be well prepared for the mentoring sessions and respectful of the mentor's busy schedule. The successful mentor must be sensitive and responsive to specific needs of the mentee that may also reflect generational differences (“millennial”), gender-based differences, or minority perspectives.

I began the American Rhinologic Society Mentorship Program after recognizing that young rhinologists in our society were earnestly seeking to connect with more experienced rhinologists, but most felt disconnected from the more seasoned generation of rhinologists in the society. To address the issue of access to mentors, we established a matching program that paired senior and junior members of the society based on professional interests, practice style and gender. Thus a young rhinologist with a basic science research interest could be paired with a senior clinician-scientist who had successfully obtained research grant funding, while a young private practice rhinologist could pair up with a more senior rhinologist in private practice who had traveled a similar path and understood the associated challenges. Each year more than 60 people participate in the program, scheduling face-to-face sessions or extended phone calls throughout the year. In offering participants the opportunity to build strong mentoring relationships early in their career, our hope is that the mentees eventually become the mentors, as they often do, which makes the program sustainable.

And lastly, it is important to remember that we can all benefit from mentorship regardless of the stage of our career. While some of the most rewarding relationships I have enjoyed professionally have been in the context of serving as a mentor, I have also continued to seek mentors myself after two decades as a practicing academic otolaryngologist. Even the mentors need mentors – being open to new perspectives and the wisdom of others can be transformative and refreshing, just as much in the later stages of one's career as in its early stages. And by experiencing high-quality mentorship, we are challenged further to be the best mentors we can be for our own mentees.

## Conflicts of interest

The author declares no conflicts of interest.
